# Outcome analysis of pelvic ring fractures

**DOI:** 10.4103/0019-5413.58610

**Published:** 2010

**Authors:** Ramesh K Sen, Lokesh A Veerappa

**Affiliations:** Department of Orthopaedic Surgery, Post Graduate Institute of Medical Education and Research, Chandigarh, India

**Keywords:** Long-term outcome, residual displacement, sacroiliac disruption, pelvic ring fracture

## Abstract

**Background:**

The behavior of pelvic ring fractures in the long run has been very sparsely studied. The purpose of this study is to assess the long-term outcome of pelvic ring fractures.

**Materials and Methods:**

A total of 24 patients with pelvic ring fractures, not involving the acetabulum, were followed up for an average duration of 33 months (range 24–49 months). The clinicoradiological assessment was done using the pelvic scoring system adapted from Cole *et al*. Parameters assessed included sacroiliac (SI) joint involvement and, among SI joint injuries, the presence of a fracture disruption and the degree of displacement.

**Results:**

Pain and limp were present in 13 patients (54.2%) each and residual working disability in 9 patients (37.5%). The overall Cole's pelvic score was 31.3 ± 7.02 of a total score of 40. The average pelvic score in patients with SI disruption was 29.2 ± 6.75; much lower than patients without SI disruption with an average score of 34.9 ± 6.25 reaching statistical significance. The pelvic score among patients with a displacement ≤10 mm was 33.0 ± 3.92 and with a displacement >10 mm 25.88 ± 7.14. The difference was statistically significant.

**Conclusions:**

Pelvic ring injuries can lead to long term problems significantly. The involvement of the SI joint affects the long-term outcome adversely, more so if the residual displacement is >10 mm. The pelvic scoring system is comprehensive and depicts subtle differences in the outcome, which the individual parameters of the assessment fail to show.

## INTRODUCTION

Pelvic fractures constitute 3% of all skeletal injuries.^1^ Among polytrauma patients the incidence is about 25%.^1^ The diagnosis, management, early morbidity and high mortality in pelvic fractures have been discussed at length. There are very few long-term studies of pelvic fractures worldwide documenting the behavior of these injuries in the long run. Henderson^2^ (1989) followed 26 patients with major pelvic disruptions and found low backache in 50%, limp in 32%, and work disability in 43% of patients. Tornetta *et al*.^3^ (1996) achieved a high long-term success rate with operative management of pelvic fractures while Miranda *et al*.^4^ found no difference in the outcome among operatively and nonoperatively managed cases of pelvic fractures.

The actual outcome is likely to be much more adversely affected with the cultural practices of sitting and squatting. The present study was hence planned to critically assess the long-term outcome in pelvic fractures to answer these uncertainties found in the previous studies and find factors influencing the long-term outcome.

## MATERIALS AND METHODS

Ninety-eight consecutive patients with pelvic ring injuries managed in between 1996 and 2002 were screened. Seventy patients with complete treatment details and X-rays were called to the outpatient department for follow-up. Of these patients, 32 attended follow-up. Among these patients, eight cases had an associated acetabular fracture. These patients were excluded from our study as these would have acted as confounding factors in the assessment of the pelvic fracture outcome. Among 24 patients with pelvic ring fractures, not involving the acetabulum, the average duration of follow-up was 31.3 months (range 24–49 months). There were 21 males and 3 females in this group. The mean age was 30 years (17–58 years).

The radiological data retrieved from the old records included an anteroposterior, inlet and outlet radiograph of the pelvis.^5^ There were 15 patients with sacroiliac (SI) disruption and nine patients without SI disruption. The cases of SI disruptions were grouped into those with a displacement less than or equal to 10 mm and those greater than 10 mm (calculated using radiographs as shown by Henderson *et al*.^2^ and McLaren *et al*.^6^). Among patients without SI disruptions, three had sacral fractures and six had anterior pelvic fractures. Overall, there were four patients with anteroposterior compression (APC), 12 with lateral compression (LC), three with vertical shear (VS) and five with combined mechanism (CM) of injury according to Young's classification.^7^ All patients with pelvic ring injuries without SI disruptions were conservatively managed with bed rest.

Among SI disruptions, there were 10 patients with a pure SI disruption and five patients with a SI fracture disruption. Of the five patients with SI fracture disruptions, two had fracture of the sacral ala and three had an iliac blade fracture. Their mean age was 28 years (range 18–58 years), average duration of follow-up was 28 months (range 24–45 months), and sex ratio (male:female) was 13:2.

These patients with SI disruptions were managed either by conservative (5 cases) or operative (10 cases) means. Conservative treatment was planned in two patients with a displacement less than 10 mm and in three patients with comorbidities precluding surgery. The conservative mode of treatment included bed rest, traction in VS and CM injuries, and sacral corset in APC injuries. All cases where surgery was performed had an initial displacement of >10 mm. Surgical treatment was with reconstruction plates or partially threaded cancellous screws, either by an iliofemoral approach (seven cases) or by a posterior approach to the SI joint (three cases). Two such fixations have been illustrated in [Figures 1 and 2].

**Figure 1a:**
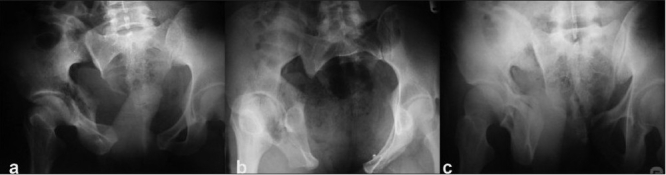
Anteroposterior (a), inlet (b), and outlet (c) views of a pelvic disruption due to a combined mechanism injury in a 27-year-old adult male patient. Note the sacroiliac disruption on the right side with pubic diastasis indicating the APC II component of the injury along with proximal migration of the hemipelvis indicating a vertical shear component

**Figure 1b:**
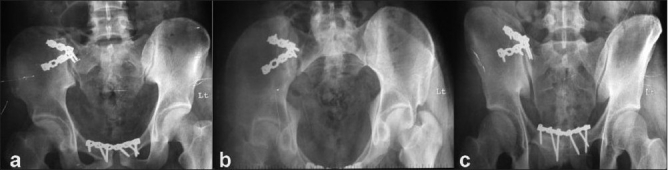
Postoperative anteroposterior (a), inlet (b), and outlet (c) radiographs showing reduction and fixation of the pelvic disruption. Note the divergent nature of the sacroiliac reconstruction plates to maximize the stability of fixation. Also, note the anterior symphyseal fixation which closes the pelvic ring

**Figure 1c:**
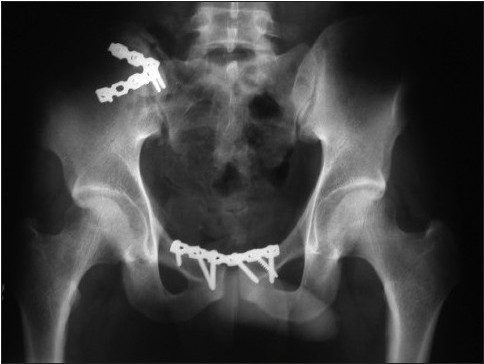
Three years postoperative radiograph of the patient showing well-maintained fixation. The patient was ambulating well without any symptoms

**Figure 2:**
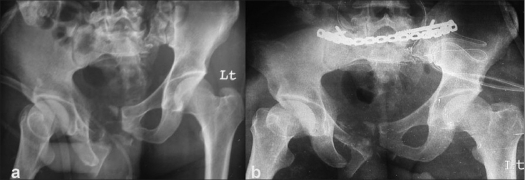
Preoperative (a) and postoperative (b) radiographs showing a vertical shear type of pelvic disruption with a zone 2 fracture of the sacrum fixed with a posterior percutaneous reconstruction plate and lag screw fixation. Note that the vertical shear component of the disruption has been addressed

These patients were assessed for the presence of individual symptoms of pain, limp, abductor function, and residual working disability. They were also assessed using Cole's pelvic score.^8^ Cole's Pelvic Scoring System is a comprehensive scoring system that incorporates several parameters assessed of history, physical examination, and radiographic evaluation. Each parameter is given a score depending on the severity. The parameters assessed during history taking include pain, narcotic use, and the ability to resume activities. The parameters assessed during physical examination include gait, Trendelenberg's test, sacral and/or pubic tenderness, lower extremity muscle group strength, and hip and trunk range of motion. Radiographically, the amount of residual displacement is assessed posteriorly and anteriorly.

The combined previous and current data were assessed statistically using SPSS 11.0 software. Pearson's chi-square test was used for the assessment of statistical significance of the presence versus absence of pain, limp, and abnormal abductor function. The Mann–Whitney test was used for the statistical assessment of the pelvic score.

## RESULTS

Among the 24 patients studied, pain and limp were present in 13 (54.2%) each. The assessment of the abductor function with Trendelenberg's test was positive in nine cases (37.5%). Fifteen of the total patients (62.5%) went back to their original jobs. Among the other nine with residual working disability (37.5%), eight quit working while one patient changed to a different job. The overall Cole's pelvic score was 31.3 ± 7.02 of a total score of 40.

The effect of SI joint involvement on the outcome is depicted in Table 1. The parameters of pain, limp and abnormal abductor function were only marginally increased on an average among the patients with SI joint involvement as compared to patients without SI joint involvement (*P* >0.05). However, the Cole's pelvic score which encompasses all these above-said parameters was significantly different among the two groups statistically. The average Cole's pelvic score in patients with SI disruption was 29.2 ± 6.75 much lower than patients without SI disruption with an average score of 34.9 ± 6.25. The difference was statistically significant (*P*-value of 0.014).

**Table 1:** The effect of sacroiliac joint involvement on outcome

Assessment parameters	Pain	Limp	Abnormal abductor function	Cole's pelvic score
SI disruption (n=15)	9 (60)	9 (60)	6 (40)	29.2 ± 6.75
No SI disruption (n=9)	4 (44.4)	4 (44.4)	3 (33.3)	34.9 ± 6.25
*P*-value	0.459	0.459	0.744	0.014*

* Significant value, , SI: Sacroiliac, Figures in parenthesis are in percentage

The parameters of pain, limp, and abnormal abductor function were present to a lesser extent in patients with pure SI disruptions than SI fracture disruptions. However, there was no statistically significant difference between pure SI disruptions and SI fracture disruptions in terms of pain, limp, or abductor function (*P*>0.05). Even the pelvic score did not show any statistically significant difference in the outcome between pure SI disruptions (29.9 ± 5.57) and SI fracture disruptions (27.8 ± 9.28).

The effect of residual displacement on the outcome in SI disruptions is depicted in Table 2. The cases of SI disruptions were grouped into those with a displacement less than or equal to 10 mm and those with a displacement greater than 10 mm.^2^,^6^ The average displacement in the former group is 5.29 ± 2.69 mm and in the latter subgroup 15.88 ± 6.49 mm. Here again the parameters of pain, limp, and abductor function did not show statistically significant difference between the two groups (*P*>0.05). However, the Cole's pelvic score among patients with a displacement less than or equal to 10 mm was 33.0 ± 3.92 and among patients with a displacement greater than 10 mm was 25.88 ± 7.14. The difference was statistically significant (*P*-value 0.036).

**Table 2:** The effect of residual displacement on the outcome in SI disruptions

Assessment parameters	Pain	Limp	Abnormal abductor function	Cole's pelvic score
Displacement ≤10 mm (n=7)	3 (42.9)	3 (42.9)	3 (42.9)	33.0 ± 3.92
Displacement >10 mm (n=8)	6 (75)	6 (75)	6 (75)	25.88 ± 7.14
*P*-value	0.205	0.205	0.205	0.036*

* Significant value, Figures in parenthesis are in percentage

Since the residual displacement was a factor that affected the outcome, an assessment was made of the cases of SI disruptions managed conservatively and operatively and the difference was not found statistically significant indicating that the line of management is of no significance as long as a good fracture reduction is achieved. This is depicted in Table 3.

**Table 3:** The effect of line of management on the outcome in SI disruptions with good fracture reduction

Assessment parameters	Cole's pelvic score
Conservative management (n=2)	31.0
Operative management (n=5)	33.3 ± 4.18
*P*-value	0.571

## DISCUSSION

An attempt to analyze the long-term problems in pelvic ring injuries has been made. Residual pain was found to be present in 54.2% (n=13) of patients. This stands in comparison to 50% with Henderson *et al*.^2^ and 46.5% with McLaren *et al*.^6^ Limp was found to be present in 54.2% (n=13) of cases in comparison to 23% with Henderson *et al*.^2^ In this study, 37.5% (n=9) ceased to work or changed to a different job after treatment. This is in comparison to 38% residual work disability with Henderson's^8^ study.

Among pelvic ring fractures, the importance of posterior injuries, especially the SI joint injury as a major factor affecting the outcome is quite explicit in the literature.^2^,^8^ In Tile's^9^ series, 40% of patients had residual pain and among these 90% had posterior lesions. Dujardin *et al*.^10^ have shown that SI disruptions behave badly in comparison to rest of the pelvic ring fractures in terms of the outcome. So in the present study, SI disruptions and non-SI disruptions were compared and a statistically significant difference in the pelvic score (*P* = 0.014) was found. This is attributable to the crucial role of SI joint in weight transmission unlike anterior injuries. In this study pain, was present in 60% (n=15) of our cases with SI disruptions as compared to 40% (n=9) in the rest of the pelvic ring fractures. Limp was also present in 60% (n=15) of cases with SI disruptions as compared to 40% (n=9) in the rest of the pelvic ring fractures. But this difference in the presence of pain and limp did not reach statistical significance due to small number of cases in this study group. Dujardin *et al*.^10^ in his study have shown that pure SI disruptions gave a worse outcome if not adequately reduced (*P*<0.001) while in SI fracture disruptions, the fracture displacement did not have a bearing on the outcome. He attributed this to fracture disruptions uniting with whatever displacement present and making pelvis stable unlike pure disruptions, which remained unstable if not reduced. In the present study, it was found that there is no significant difference between the outcome scores (*P*>0.05) of pure SI disruptions and SI fracture disruptions. This may be attributable to the small number of patients (n=5) in the SI fracture disruption subgroup and the pure SI disruption group (n=10).

Several studies have echoed the importance of residual displacement in SI disruptions in terms of long-term outcomes. Henderson *et al*.^2^ have shown that 100% of their patients with severe residual displacement had pain in comparison to 18% without displacement and 56% with slight displacement. McLaren *et al*.^6^ have shown that 12% of their patients with a ≤ 10 mm displacement had pain in comparison to 92% with a >10 mm displacement. In this study, 43% of patients with a ≤ 10 mm displacement had pain while 75% of patients with a >10 mm displacement had pain.

Henderson *et al*.^2^ have shown that 17% of their patients without vertical displacement had shuffling gait in comparison to 28% with slight residual displacement and 60% with severe displacement. In the present study, it was found that 42% of the patients with a ≤ 10 mm displacement had a limp in comparison to 75% with a > 10 mm displacement [Table 2].

When the displacement was taken as the criteria and assessed, a statistically significant difference was noticed in the pelvic score (*P* = 0.036) indicating the importance of achieving at least less than or equal to 1 cm of displacement for a better long-term outcome. However, the line of management had no significance as long as a good reduction of ≤ 10 mm was achieved.

Our study reiterates the authenticity of the pelvic scoring system, which is comprehensive and effective in depicting differences that individual parameters like pain or limp fail to show.

In conclusion, posterior injuries especially the SI disruptions affect the long-term outcome significantly and if a good reduction can be achieved by any line of management, we can expect a good outcome. Reducibility is the most important factor in the outcome of these injuries.
